# The Neglected Tropical Diseases of Latin America and the Caribbean: A Review of Disease Burden and Distribution and a Roadmap for Control and Elimination

**DOI:** 10.1371/journal.pntd.0000300

**Published:** 2008-09-24

**Authors:** Peter J. Hotez, Maria Elena Bottazzi, Carlos Franco-Paredes, Steven K. Ault, Mirta Roses Periago

**Affiliations:** 1 Department of Microbiology, Immunology, and Tropical Medicine, The George Washington University and Sabin Vaccine Institute, Washington, D.C., United States of America; 2 Hospital Infantil de México, Federico Gómez, México, D.F., México; 3 Department of Medicine, Emory University, Atlanta, Georgia, United States of America; 4 Pan American Health Organization/World Health Organization (PAHO/WHO), Washington, D.C., United States of America; Centers for Disease Control and Prevention, United States of America

## Abstract

The neglected tropical diseases (NTDs) represent some of the most common infections of the poorest people living in the Latin American and Caribbean region (LAC). Because they primarily afflict the disenfranchised poor as well as selected indigenous populations and people of African descent, the NTDs in LAC are largely forgotten diseases even though their collective disease burden may exceed better known conditions such as of HIV/AIDS, tuberculosis, or malaria. Based on their prevalence and healthy life years lost from disability, hookworm infection, other soil-transmitted helminth infections, and Chagas disease are the most important NTDs in LAC, followed by dengue, schistosomiasis, leishmaniasis, trachoma, leprosy, and lymphatic filariasis. On the other hand, for some important NTDs, such as leptospirosis and cysticercosis, complete disease burden estimates are not available. The NTDs in LAC geographically concentrate in 11 different sub-regions, each with a distinctive human and environmental ecology. In the coming years, schistosomiasis could be eliminated in the Caribbean and transmission of lymphatic filariasis and onchocerciasis could be eliminated in Latin America. However, the highest disease burden NTDs, such as Chagas disease, soil-transmitted helminth infections, and hookworm and schistosomiasis co-infections, may first require scale-up of existing resources or the development of new control tools in order to achieve control or elimination. Ultimately, the roadmap for the control and elimination of the more widespread NTDs will require an inter-sectoral approach that bridges public health, social services, and environmental interventions.

## The Neglected Tropical Diseases in the Latin American and the Caribbean Region

The neglected tropical diseases (NTDs), a group of chronic, debilitating, and poverty-promoting parasitic, bacterial, and some viral and fungal infections, are among the most common causes of illness of the poorest people living in developing countries [1]. Their control and elimination is now recognized as a priority for achieving United Nations Millennium Development Goals (MDGs) and targets for sustainable poverty reduction [1]–[3]. Approximately 40% of the estimated 556 million people living in the Latin American and the Caribbean region (LAC) live below the poverty line, including 47 million people who live on less than US$1 per day, and another 74 million people who live on less than US$2 per day [4],[5]. Relative to sub-Saharan Africa and Asia, where NTDs also occur, the character of poverty in LAC is unique. In terms of income distribution, LAC exhibits the highest inequality anywhere [6], with the richest one-tenth of the population earning 48% of total income and the poorest tenth earning only 1.6% [7]. Of LAC's estimated 213 million impoverished people, approximately one-third live in rural poverty as subsistence farmers, ranchers, and fishermen [8], typically in communities of indigenous and African descent where they face a high level of social exclusion and social inequity [9], including lack of access to safe water and health care services [10],[11]. Two-thirds of the region's poor live in *favelas*, *asentamientos pobres*, *barrios pobres*, *turgurias*, and *áreas periféricas*, i.e., urban and peri-urban communities where poverty combines with the conditions of unsafe water, poor sanitation, and the proliferation of rodent animal reservoirs and vectors [8],[12].

Poverty is not the only major determinant for risk of acquiring NTDs in LAC. Instead, it combines with other inequities related to ethnicity (e.g., indigenous groups and people of African descent), age and gender (i.e., children and women), and a patchwork of unique ecological niches to establish sometimes highly focal epidemiological NTD “hot spots.” This has important implications for the control of NTDs in LAC, which may differ from the integrated NTD control currently being advocated for and tested in sub-Saharan Africa and elsewhere [1]. Here, we focus on some of the unique aspects of NTD disease burden and endemicity in the LAC region, as well as the prospects for NTD control and elimination in the region. The review of the literature was conducted using the online database PubMed from 2000 to 2007 with United States National Library of Medicine Medical Subject Headings, the specific diseases listed as neglected tropical diseases on the *PLoS Neglected Tropical Diseases* Web site (http://www.plosntds.org), and the geographic regions and countries of LAC. Reference lists of identified articles and reviews were also hand searched as were databases from the Pan American Health Organization (PAHO) Web site (http://www.paho.org).

## Burden and Geographic Distribution of Disease

The NTDs in LAC may be characterized by two major patterns of disease distribution. The first is a pattern of widespread endemicity such as that seen for the soil-transmitted helminth (STH) infections, Chagas disease, and dengue; the second pattern is one of geographically restricted endemicity as the result of concerted public health interventions and ecological conditions as seen for onchocerciasis, lymphatic filariasis (LF), and schistosomiasis in areas such as the Caribbean and Guyana shield. The latter group may represent a distinct situation from other parts of the world, as they may be said to represent a “last stronghold” of endemic focal communicable diseases, that can be eliminated in a region but are not yet. In this sense, the presence of such NTDs represents a moral burden as well as an epidemiological burden. Because they are seen by some as illustrations of the failure of primary health care implementation [13], the NTDs also represent a moral imperative for action to complete primary health care implementation where it has failed and make it accessible to all.

Ault [8] previously listed the major NTDs in LAC, and Table 1 ranks these NTDs by their estimated prevalence, at-risk population, and distribution, while Tables 2 and 3 rank the NTDs by their estimated disease burdens measured in disability-adjusted life years (DALYs). The major STH infections are the most prevalent NTDs, and the STH infections and Chagas disease are responsible for the highest estimated NTD burden in LAC. They are followed by dengue, schistosomiasis, leishmaniasis, and other NTDs. However, dengue is considered underreported in the LAC region [14], and because leishmaniasis frequently occurs in remote areas or regions of guerilla conflict [15], its disease burden is not well established except in some areas of Brazil, Peru, and Bolivia [16]. Based on global disease burden estimates in DALYs published previously by the World Health Organization (WHO) and other investigators [17],[18], a range of estimates for NTDs in LAC is provided in Table 2. Such DALY estimates were obtained by adjusting the global estimates according to the percentage of the disease burden determined to occur in LAC, or in some cases they were quoted directly from WHO estimates (Table 2). From this analysis it was determined that the total burden of these NTDs in LAC may exceed the disease burdens from malaria or tuberculosis, and according to some estimates, the regional NTD disease burden exceeds that of HIV/AIDS (Table 2). There are also a number of other important NTDs in LAC (many of which are zoonoses) for which the disease burdens as expressed in DALYs have not been determined or reported (Table 3).

**Table 1:** Ranking of NTDs in LAC by Prevalence and Distribution.

Disease	Population Currently Infected in LAC	Population At Risk in LAC	Major Vulnerable Populations or Geographic Areas	Number LAC Countries Infected	Percentage of LAC Population Infected (% Poor People Infected)	Percent Global Disease Burden in LAC	Reference
Trichuriasis	100 million	523 million	Poor rural & urban slums	27	17.8% (46.9%)	16.6%	[19]
Ascariasis	84 million	514 million	Poor rural & urban slums	27	15.0% (39.4%)	10.4%	[19]
Hookworm	50 million	346 million	Poor rural	26	8.9% (23.5%)	8.7%	[19]
Chagas disease	8–9 million	25–90 million	Poor rural & urban slums	13	1.6% (4.1%)	99.8%	[43],[44]
Schistosomiasis	1.8 million	36 million	Poor rural	4 with >1,000 cases	0.3% (0.8%)	0.9%	[31]
Blinding trachoma	1.1 million	ND	Poor rural	3	0.2% (0.5%)	1.3%	[54]
Lymphatic filariasis	720,000	8.9 million	Urban slums & poor rural	7	0.1% (0.3%)	0.6%	[20],[33]
Dengue	552,141 reported in 2006	ND	Urban slums	23	0.1% (0.2%)	ND	[62]
Cysticercosis	400,000	75 million	Poor rural	15	<0.1% (0.2%)	ND	[37]
Cutaneous (CL) and visceral (VL) leishmaniasis	62,000 CL	ND	Urban slums & poor rural	18	ND	ND	[52]
	5,000 VL						
Leprosy	47,612 new cases	ND	Poor rural & urban slums	22	<0.1% (<0.1%)	11.4%	[56]
Onchocerciasis	64 new cases in 2004	515,675	Poor rural	6	<0.1% (<0.1%)	0.3%	[20],[35],[36]
Jungle yellow fever	86 new cases in 2004	ND	Jungle & urban slums	4	<0.1% (<0.1%)	<0.1%	[63]

ND, not determined.

**Table 2:** Ranking of NTDs by Disease Burden (DALYs) and Comparison with HIV/AIDS, Tuberculosis, and Malaria.

Disease	Estimated Global Disease Burden in DALYs	Number of Cases in LAC (Number of Cases Worldwide)	Estimated Percentage of Disease Burden in LAC	Estimated LAC Disease Burden in DALYs	Reference
Hookworm infection	1.5–22.1 million	50 million (576 million)	8.7%	130,500–1,923,000	[17],[75]
Ascariasis	1.2–10.5 million	84 million (807 million)	10.4%	124,800–1,092,000	[17],[75]
Trichuriasis	1.6–6.4 million	100 million (604 million)	16.6%	265,600–1.062,000	[17],[75]
Chagas disease	0.667 million	ND	99.8%	662,000	[18]
Dengue and DHF	0.6 million	ND	11.2%	69,000	[18]
Leishmaniasis	2.1 million	ND	2.1%	44,000	[18]
Schistosomiasis	4.5 million	1.8 million (207 million)	0.8%	36,000	[17],[31]
Lymphatic Filariasis	5.8 million	0.72 million (120 million)	0.6%	34,800	[18],[33]
Trachoma	2.3 million	1.1 million (84 million)	1.3%	23,200	[18],[54]
Leprosy	0.2 million	ND	9.0%	18,000	[18]
**Total NTDs**	**56.6 million**		**8.8%**	**1,407,900–4,964,000**	
HIV/AIDS	84.5 million		3.8%	3,211,000	[18]
Tuberculosis	34.7 million		2.7%	928,000	[18]
Malaria	46.5 million		0.2%	111,000	[18]

DALYs for each disease in LAC were calculated using global burden data in [17] and [18] and followed by a determination of the percentage of the disease burden in LAC based on the estimated number of cases in LAC (Table 1) divided by the estimated number of cases worldwide [1] multiplied by 100. Alternatively, for Chagas disease, dengue and dengue hemorrhagic fever (DHF), leishmaniasis, and leprosy, information from the disease burdens quoted in [18] were used.

**Table 3:** Major NTDs with No National or Regional Disease Burden Estimates in DALYs.

Helminth Infections	Protozoan Infections	Bacterial Infections	Fungal Infections and Ectoparasitic Infections	Viral Infections
Echinococcosis	Amebiasis	Bartonellosis	Mycetomas	Hemorrhagic fevers
Cysticercosis	Giardiasis	Buruli ulcer	Paracoccidioidomycosis	Rabies
Fascioliasis		Leptospirosis	Myiasis	
Strongyloidiasis		Plague	Scabies	
Toxocariasis		Treponematoses (non-venereal)	Tungiasis	

### 

#### Helminth infections

The major helminth infections in LAC include *Necator americanus* hookworm infection and other STH infections, schistosomiasis, LF, cysticercosis, and onchocerciasis. Trichuriasis (100 million cases) and ascariasis (84 million cases) are the most prevalent NTDs and widely distributed throughout LAC [19] (Table 1), with the largest estimated number of cases in Brazil, Mexico, and Guatemala (Table 4). Guatemala exhibits the highest prevalence of trichuriasis and ascariasis [19], which may partly explain why this nation has the highest prevalence of underweight children [20]; high rates of these two infections also occur along the north Pacific coast of South America, where they are associated with growth stunting [21], as well as in other parts of Central America and the Caribbean [19]. By some estimates, hookworm is the single leading cause of disease burden among the NTDs (Table 2). Of the 50 million cases of hookworm infection that occur in poor rural areas, approximately 65% occur in Brazil (Table 4) [19]. In some regions of Minas Gerais State, Brazil, an estimated 68% of the rural population is infected with hookworm [22], where it is a major cause of anemia in children [23]. High rates of infection also occur in neighboring Paraguay and Peru [19],[21],[24],[25], as well as in parts of Central America and in Suriname [19]. Hookworm is also a major cause of adverse pregnancy outcomes in LAC [25]. Two other STH infections, toxocariasis [26],[27] and strongyloidiasis [28],[29], are also endemic in LAC, but there are no estimates of their regional prevalence.

In 2000, Chitsulo et al. [30] determined that almost all of the estimated 7.3 million cases of schistosomiasis in LAC, caused exclusively by *Schistosoma mansoni*, occur in Brazil. More recently, Steinmann et al. [31] estimated that there are currently only 1.8 million cases in LAC, with 84% of the cases in Brazil (Table 4). The largest number of cases occurs in the eastern Brazilian states of Minas Gerais and Bahia, as well as in the small neighboring northeastern states of Sergipe, Alagoas, Pernambuco, and Paraiba [32]. In Brazil, there is a high degree of *N. americanus* and *S. mansoni* co-infection [22]. Outside of Brazil, *S. mansoni* infection occurs in the Caribbean, especially in the Dominican Republic and in Venezuela and Suriname [31].

**Table 4:** Geographic Distribution and Estimated Burden of the Major Helminthiases in LAC.

Disease	Total Number of Cases	Country (Greatest Number of Cases)	Country (2nd Greatest Number of Cases)	Country (3rd Greatest Number of Cases)	Country (4th and 5th Greatest Number of Cases)	Reference
Trichuriasis	100 million	Brazil	Mexico	Colombia	Guatemala	[19]
		18.9 million	18.3 million	15.4 million	8.6 million	
					Venezuela	
					8.7 million	
Ascariasis	84 million	Brazil	Mexico	Guatemala	Argentina	[19]
		41.7 million	9.3 million	7.9 million	7.7 million	
Hookworm	50 million	Brazil	Paraguay	Guatemala	Colombia	[19]
		32.3 million	3.2 million	3.0 million	3.0 million	
Schistosomiasis	1.8 million	Brazil	Dominican Republic	Venezuela	Guadeloupe	[31]
		1.5 million	258,000	23,674	4,400	
					Suriname	
					3,935	
Lymphatic filariasis	0.72 million	Haiti	Brazil	Dominican Republic	Guyana	[20],[33]
		560,000	60,000	50,000	50,000	
	8.9 million at risk	6.0 million at risk	1.5 million at risk	0.74 million at risk	0.63 million at risk	
Onchocerciasis	0.52 million at risk	Guatemala	Mexico	Venezuela	Ecuador	[20],[35],[36]
		0.20 million at risk	0.17 million at risk	0.11 million at risk	0.02 million at risk	

Currently, four countries—Brazil, Dominican Republic, Guyana, and Haiti—report active transmission of LF and are actively conducting control or elimination efforts through mass drug administration (MDA) [33]. Almost 80% of the LF cases occur in Haiti, where more than 70% of that nation's population is also at risk for infection [20],[33]. In Brazil, LF occurs primarily in the metropolitan areas of Recife (Pernambuco State) and Maceio (Alogoas State) [33]. Through MDA, campaigns to eliminate LF (2.4 million people were treated in 2007) in the Caribbean are in progress [33],[34]. Onchocerciasis is currently endemic in six countries, Brazil, Colombia, Ecuador, Guatemala, Mexico, and Venezuela [35]. Through the Onchocerciasis Elimination Program for the Americas (OEPA), more than 85% of eligible populations in 13 endemic foci have been receiving ivermectin MDA [35]. Foci in Guatemala, southern Mexico, and Venezuela have the highest percentage of the population needing MDA [35]. OEPA has now come close to ending onchocerciasis ocular morbidity in the Americas [35], and has established guidelines in coordination with the WHO for the certification of onchocerciasis elimination [36]. Transmission has been declared interrupted in two foci since 2007, one in Colombia and one in Guatemala.

The major platyhelminth infections in LAC are three zoonoses: cysticercosis, fascioliasis, and paragonimiasis. There are an estimated 400,000 people with symptomatic cysticercosis in LAC [37]. The infection may be eliminatable through better pig husbandry and/or MDA of pigs and humans [37]. Human fascioliasis is an important sheep-associated zoonosis in the Chaco of Bolivia, Paraguay, and Argentina, in the Andean highland region (the Altiplano [38],[39]), and in parts of the Caribbean, including Cuba, Dominican Republic, and Haiti [40],[41]. Paragonimiasis has been reported from Colombia, Ecuador, and Mexico [42], while echinococcosis is another major zoonosis in areas dependent on sheep and other livestock [20].

#### Protozoan infections

Chagas disease is one of the highest disease burden NTDs in LAC [43]–[50]. Almost all of the 8–9 million cases of Chagas disease [43],[44] (with approximately 50,000 new cases annually [44]) occur in poor rural and, increasingly, many new urban and peri-urban areas of Latin America (Table 1). It is estitamed that up to 5.4 million people will develop chronic Chagas heart disease [20],[45], while 900,000 will develop megaesophagus and megacolon [20]. In LAC, the burden of disease caused by *Trypanosoma cruzi* infection is between five and ten times greater than malaria [46]. Moreover, its economic impact represents a significant percentage of the external debt of the region [46]. Because of the propensity of the kissing bug vector (especially *Triatoma infestans*) to live in the cracks and crevices and roofs of poor-quality dwellings, and the lack of essential medicines for patients during the acute stages of infection, Chagas disease is disproportionately represented among people living in poverty [12],[46]. Despite successful elimination efforts in the southern cone of South America [50] (for reasons discussed below), the disease remains endemic in many regions of Central and South America [48],[49]. Chagas disease has also emerged or re-emerged in areas of conflict, including Chiapas State, Mexico [47], and Colombia [15]. Increasingly, dogs are recognized as important animal reservoirs of the infection [48]. The major approaches to control include improved case management and vector control programs, together with housing improvement through regional programs, which have been reviewed previously [20], [48]–[50].

In LAC, both cutaneous and visceral forms of leishmaniasis result primarily from zoonotic transmission from either canine or sylvatic (e.g., opossum, sloth, anteater) reservoir hosts. The most important determinants for the emergence of both new world zoonotic cutaneous leishmaniasis (ZCL) and zoonotic visceral leishmaniasis (ZVL) include poverty, urbanization, and human migration [16]. *Leishmania mexicana*, *L. amazonensis*, *L. braziliensis*, *L panamensis*, *L. peruviana*, and *L. guyanensis* are the major species that cause new world ZCL [51]. Approximately 62,000 cases of ZCL occur primarily in Brazil, Colombia, Paraguay, Venezuela, Panama, Ecuador, and Peru [52] (Table 5), where urbanization near *Lutzomyia* sandfly breeding sites has led to an increase in the number of cases [53]. In addition, the emergence of ZCL in Colombia is linked to several decades of armed and guerilla internal conflict fueled by cocaine production and trafficking [15]. In northeastern Brazil, ZVL (*L. chagasi*) has become an important infection in the *favelas* of Forteleza, Salvador do Bahia, and other urban centers, including Rio de Janeiro and Belo Horizonte [53]. In these impoverished urban and peri-urban settings, the cracked walls and damp earth floors, together with an absence of sanitation and inadequate garbage collection, combine to create sandfly breeding sites [16]. With the exception of Brazil, surveillance systems in Latin America have been limited in their capacity to assess the true burden of ZVL. A regional leishmaniasis control action plan is now being implemented [20].

**Table 5:** Geographic Distribution and Estimated Burden of Cutaneous Leishmaniasis and Visceral Leishmaniasis in LAC.

Disease	Total Number of Cases	Cases by Country						Reference
		Brazil	Colombia	Peru		Nicaragua	Bolivia	
Leishmaniasis	62,000 CL	28,375 CL	22,000 CL (2005)	7,127 CL (2005)	3,312 CL (2005)		2,800 CL (2004)	[20],[52]
	5,000 VL	3,386 VL (2004)						

Some estimates are from 2004, others from 2005.

CL, cutaneous leishmaniasis; VL, visceral leishmaniasis.

#### Bacterial and fungal infections

The most important bacterial NTDs are trachoma, leprosy, and some of the bacterial zoonoses, especially leptospirosis. There are approximately 1 million cases of trachoma (ocular *Chlamydia trachomitis* infection) in Latin America (Table 1), with 97% of the cases in Brazil and the remainder in Guatemala and Mexico [54] (Table 6). Although overall trachoma is not considered a major cause of blindness in LAC [20], the Amazonian region is severely affected, and some indigenous school-aged populations exhibit prevalence as high as 42% [55]. A federal school-based program for the control and antibiotic treatment of trachoma is underway in Brazil. In Central America, trachoma is endemic in focal areas of Guatemala [55], while in Chiapas State, Mexico, the disease is on the verge of elimination [20]. A total of 64,715 cases of leprosy (*Mycobacterium leprae*) were in treatment in LAC with multi-drug therapy in 2006, with 47,612 new cases detected (Table 1) [56]. Brazil has the largest leprosy disease burden in LAC with 93% of the new cases, and it is the only LAC country that has not yet achieved a goal to eliminate leprosy below the target of one case per 10,000 population [20]. Most of the cases of human brucellosis go undiagnosed or unreported in LAC, while bovine tuberculosis has been eliminated in many regions [20]. Leptospirosis is also an important cause of morbidity in LAC, especially in the *favelas* of Brazil and other urban slums [10],[57],[58], where it has been linked to a very serious pulmonary hemorrhage syndrome [58]. Bartonellosis remains an important local sandfly-transmitted bacterial infection in the Andean region [59], while cases of Buruli ulcer are reported occasionally in LAC. Several mycoses, such as paracoccidioidomycosis and mycetoma, are responsible for major public health and economic hardships in Latin America; these tend to concentrate around humid forests in subtropical and tropical areas [60],[61].

**Table 6:** Geographic Distribution and Estimated Burden of the Bacterial NTDs in LAC.

Disease	Total Number of Cases	Cases by Country				Reference
Blinding trachoma	1.1 million cases (2003)	Brazil	Guatemala	Mexico		[54] a
		1,064,218 (2003)	2,073 (2003)	290 (2003)		
Leprosy	47,612 new cases (2006)	Brazil	Venezuela	Paraguay	Colombia	[56]
		44,436 (2006)	768 (2006)	404 (2006)	398 (2006)	

a The number of cases of trachoma in the LAC region was determined by querying the WHO global health atlas, selecting the terms noncommunicable diseases, blindness, trachoma, active trachoma (TF/TI), all ages, year, applied time period: 2003. The number of cases reported included 1,064,218 in Brazil, 2,073 in Guatemala, and 290 in Mexico.

#### Viral infections

The most important viral NTDs are dengue and yellow fevers. In 2006, more than one-half million cases of dengue fever (”classic dengue”) (Table 1) were reported, as well as 14,459 cases of dengue hemorrhagic fever (DHF) [62]. Brazil recorded the highest number of cases with 63% of LAC's dengue disease burden (Table 7); however, based on seroprevalence studies, it is believed that the number of reported cases represents only a fraction of the total number [14]. Dengue incidence is on the rise as a consequence of an increasing distribution of the vector *Aedes aegypti* (as well as a second vector, *A. albopictus*) as a result of urbanization, increased human migrations and air travel, flooding from global warming, and serious public health lapses in effective vector containment [14]. The increase in cases has been particularly striking during the 1990s and shortly after 2000, when at least 25 countries reported either epidemics or sporadic cases of DHF [14]. American dengue and DHF have a number of unique features compared to dengue in Asia, including its predilection to strike adults and children, its impact on the elderly, and several unusual clinical sequelae, including shock in the absence of hemorrhagic complications [14]. The economic impact of dengue may run into the hundreds of millions of dollars [14]. Through DENGUE-NET, a regional program for epidemiological surveillance coordinated by PAHO and the LAC ministries of health, efforts are in place to improve the reporting of statistical data [20]. Important jungle yellow fever outbreaks have been reported recently, with most of the cases in Peru in 2006 [63], in Brazil in 2007–2008, and in Paraguay and Argentina in 2008 [64],[65]. The observation of apparent urban transmission by *A. aegypti* in Paraguay in early 2008 would represent the first urban transmission seen in five decades in LAC. As a result, these countries have stepped up community and traveler vaccination campaigns and vector control and implemented a syndromic surveillance system [63], while since 2000, Peru, Bolivia, Paraguay, Colombia, Venezuela, Guyana, and Trinidad and Tobago have incorporated yellow fever vaccine into national child immunization schemes, seeking coverage rates comparable to their current measles vaccination rates [63]. The number of cases of rabies transmitted by dogs to humans continues to decline in LAC, with the majority now being reported from low-income groups living in urban slums of large cities in Bolivia, Brazil, El Salvador, and Haiti [20].

**Table 7:** Geographic Distribution and Estimated Burden of Reported Dengue Cases in LAC in 2006.

Disease	Total Number of Reported Cases	Reported Cases by Country				Reference
		Brazil	Venezuela	Colombia	Mexico	
Dengue	552,141	346,471	39,860	36,471	27,287	[62]

## The NTD-Vulnerable Populations: Peoples of Indigenous and African Descent

The NTDs in the Americas are concentrated not only within pockets of intense poverty, but also among selected vulnerable populations, especially some indigenous populations and communities of African descent. In LAC, it is estimated that 7% of the total population and 40% of the rural population belong to a unique ethnic group [20]. Rural poverty disproportionately affects indigenous people, particularly in Bolivia, Colombia, Ecuador, Guatemala, Mexico, and Peru, where 80% of these populations live [9]. In Guatemala and in the neighboring states of southern Mexico, the indigenous populations suffer from some of the highest rates of STH infection in the Americas [19], as well high rates of onchocerciasis [35] and Chagas disease [48]. Some of the indigenous populations acquire their infections in agricultural labor camps and on plantations [9],[66]. Similarly, the indigenous people of Bolivia and Peru experience high rates of fascioliasis, cysticercosis, and plague [20],[39],[67],[68]; those in Colombia are at risk for leishmaniasis, Chagas disease, and yellow fever [15]; and in Brazil, there are several well-documented examples of high levels of STH infection and subsequent growth stunting among indigenous people [69]–[72], as well as trachoma [55]. Indigenous people also often bear the brunt of vector-borne NTDs that emerge during conflict [15],[48]. In addition to LAC's indigenous communities, poor populations in communities of African descent, such as those found in parts of the Caribbean, Central America, and Brazil, suffer from high prevalence rates of NTDs, especially *N. americanus* hookworm infection, LF, onchocerciasis, and schistosomiasis. These infections were introduced into the region during the Middle Passage, so that their prevalence among the poor represents a tragic living legacy of the Atlantic slave trade [73].

## Past Successes and Current Challenges

There have been some extraordinary successes in both national and regional efforts to take measures for controlling several of the most important NTDs in LAC. First among them has been great progress towards the elimination of LF and onchocerciasis. With respect to the former, Brazil has reduced LF transmission from 11 known foci to one to two small areas, and the at-risk populations in the Caribbean region, particularly in Haiti and Dominican Republic, are receiving MDA [20],[33],[34]. Similarly, all six onchocerciasis-endemic countries have met their full treatment goals and no new ocular disease has been found in recent years; MDA with ivermectin continues in the foci with active transmission [35],[36]. In addition, the prevalence of both trachoma and leprosy has been declining in the region in recent decades [20] and there is optimism that these two ancient scourges could be eliminated in the coming decade. In the Caribbean, the incidence of schistosomiasis has been dramatically reduced [31],[32] and the disease seems potentially eliminatable. Through expanded use of insecticides, improved housing, and other interventions, great gains have been made by Iniciativa de Salud del Cono Sur (INCOSUR) in their efforts to eliminate Chagas disease from South America's southern cone [50]. An exciting new effort to eliminate Chagas disease throughout the region by 2010 has been launched through a new Global Network for Chagas Elimination [43]. Some countries, including Argentina, Belize, Ecuador, Haiti, Honduras, and Nicaragua, have recently initiated major expansions of their STH control programs.

At the same time, enormous challenges to NTD control remain. There is a need to complete elimination efforts for schistosomiasis in the Caribbean [74], and to eliminate the transmission of LF, onchocerciasis, and trachoma in Latin America. Control or elimination of the highest burden NTDs, e.g., Chagas disease, STH infections, and hookworm and schistosomiasis co-infections, still requires intensified efforts. Chagas disease remains one of the region's most devastating NTDs, and even in the southern cone where domestic transmission has been nearly eliminated through vector control of *T. infestans*, there are concerns about emerging insecticide resistance [48], or the possibility that the vacant niches will be eventually be occupied by other triatomine vectors [50]. In the Chaco, elimination of *T. infestans* vectors has not been achieved [48],[49], while in Mexico, Central America, the northern tropical regions of South America, and elsewhere, elimination efforts have been thwarted by sylvatic *T. dimidiata* vectors, which can re-invade dwellings following the use of insecticides [50]. For the case management and treatment of both Chagas disease and leishmaniasis, the major drugs used are either expensive or toxic or both, and frequently require long periods of supervised therapy [50]. There is an urgent need for developing safer anti-Chagas drug regimens and more accurate diagnostic tools to assess the efficacy of anti-trypanosomal drugs, particularly during the chronic phase of the disease [48]. Hookworm infection and other STH infections remain highly prevalent, especially in Brazil, where co-endemic hookworm infection and schistosomiasis (and hookworm and schistosomiasis co-infections) account for large-scale disability and lost economic productivity [22],[23],[75],[76]. Overall, the nation of Brazil accounts for the highest NTD burden in the Americas, and even though Brazil is also the largest country in LAC, its NTD burden is disproportionately high [77]. In addition to high rates of hookworm and schistosomiasis, Brazil also has the greatest number of cases of leishmaniasis, leprosy, and leptospirosis [77]. Also of concern are the five priority NTD-endemic countries, Bolivia, Guyana, Haiti, Honduras, and Nicaragua, targeted by PAHO for accelerated technical cooperation [8].

## Approaches to Control or Elimination of the NTDs in LAC

In sub-Saharan Africa an important approach to NTD control relies on the concept of integration and the simultaneous targeting of the most highly prevalent NTDs, i.e., ascariasis, trichuriasis, hookworm infection, schistosomiasis, LF, onchocerciasis, and trachoma, through MDA with a “rapid-impact” package of drugs [1]. In most of LAC, however, the distribution of the NTDs is not as widespread and therefore not always amenable to the same African control strategies. With the exceptions of some areas of eastern Brazil where STH and schistosome infections are also co-endemic with LF (Pernambuco and Alagoas States), and in the Amazonian basin where, particularly among indigenous people, STH infections overlap with onchocerciasis and trachoma (northern Brazil), there are limited opportunities to administer a full rapid-impact package in the Americas. Instead, the pattern of NTD endemicity in the most impoverished areas of LAC has a unique regional character, typically with STH infections or Chagas disease, the most widespread NTDs, co-endemic with a few other NTDs, especially zoonotic NTDs.

As shown in Table 8, at least 11 different sub-regions with unique human and environmental ecologies that promote NTDs have been initially identified in LAC. The regional sociodemographic character of LAC's NTDs include high prevalence in the densely populated and forgotten urban slums and highly concentrated pockets of intense rural poverty characterized by poor or no access to basic services, such as safe water and sanitation, electricity, schooling, and health care, where both human-derived and environmental factors promote NTD transmission. Equally important are the unique geographies of areas such as the dry and cold Altiplano, the dry and barren Chaco, the isolated Central America highlands, and parts of the Amazonian and Caribbean basins, each representing NTD “hot spots” where marginalized and often impoverished populations of indigenous people or people of African descent live in great poverty. For example, some indigenous communities in the Amazonian basin suffer simultaneously from STH infections, onchocerciasis, cutaneous leishmaniasis, scabies, tungiasis, and mycoses. Intense human migrations in the region because of mining, urbanization, deforestation, desertification, and armed conflict represent additional external factors that promote NTD transmission [12],[48],[78],[79].

**Table 8:** Major NTD Target Sub-Regions and Unique Ecologies.

Scenario	Sub-Region	NTDs	Indigenous Populations	Co-Factors^a^	Health Services Coverage
1	Southern cone of South America	Chagas, leishmaniasis, cysticercosis, echinococcosis, hemorrhagic fevers	+	Cattle ranching, minifundios, urban migration	++++
2	Chaco (Bolivia, Paraguay, Argentina)	Chagas, leishmaniasis, STH	+++	Cattle ranching, minifundios, animal husbandry	++
3	Andean region (Altiplano or Highland)	Fascioliasis, Chagas, leishmaniasis, plague, bartonellosis, STH, cysticercosis, echinococcosis, ectoparasites	++++	Minifundios, urban migration	++
4	Amazonian basin	Chagas, leishmaniasis, STH, onchocerciasis, leprosy, trachoma, ectoparasites	++	Deforestation, mining, guerillas, urban migration, indiscriminant colonization	+
5	Eastern Brazil	STH (esp. hookworm) schistosomiasis, Chagas disease, leishmaniasis, LF (NE only), echinococcosis, leprosy, leptospirosis	++	Cattle ranching, deforestation, minifundios, urban migration, monoculture	+
6	North Pacific of South America	STH, cystiercosis, leishmaniasis, onchocerciasis, echinococcosis	++	Deforestation, gold mining, guerillas	++
7	Caribbean basin	STH, schistosomiasis, LF, leprosy, leptospirosis, fascioliasis	+	Economic dependence on tourism, deforestation, urban migration	++++
8	Central America and Panama	STH, leishmaniasis, Chagas, onchocerciasis, cysticercosis, leptospirosis	+++	Deforestation, desertification, migration	++
10	South and Central Mexico	STH, Chagas, cystiercosis, leishmaniasis, trachoma, onchocerciasis	+++	Deforestation, migration	++
11	Northern Mexico	STH, Chagas, cysticercosis, leishmaniasis	++	Desertification, migration	++

a All sub-regions have co-factors of poor housing and lack of safe water and basic sanitation.

All of these settings are characterized by poor housing and lack of safe water and basic sanitation as co-factors of transmission. Within such settings, all three major STH infections are nearly ubiquitous among preschool and school-aged children, while hookworm infection is also common in pregnant women. Co-endemic with the STH infections are combinations of one or more of the following NTD infections: schistosomiasis (particularly in eastern Brazil), the vector-borne filarial diseases LF (Caribbean, northeastern Brazil) and onchocerciasis (northern Pacific of South America, Central America, and southern Mexico), leishmaniasis (in all sub-regions except the Caribbean), and other zoonotic NTDs. Similarly, in many of the poorest sub-regions, Chagas disease remains highly endemic. Possibly, for more than any other NTD, the knowledge gaps for Chagas disease remain the greatest, particularly with respect to the extent of zoonotic transmission from dogs and other animals, the emergence and re-emergence of triatomine vectors, the role of bednets, and the lack of specific tools for case management [48]. In addition, the extent of co-infections with Chagas disease and the other major NTDs is not well established. As summarized in Table 9, several different modalities are required to control or eliminate the unique NTDs in these 11 sub-regions, including MDA, targeted administration together with intensified early case detection and management, integrated vector management, control of animal reservoirs, behavioral interventions, and other specialized measures [8].

**Table 9:** Major Approaches to NTD Control in LAC.

Approach	Objective(s)	Diseases	Additional Control Tools under Development
1. MDA	Eliminate as a public health problem	LF, onchocerciasis	Improved diagnostics for onchocerciasis.
2. MDA and improved case detection and management	Eliminate as a public health problem	Trachoma, leprosy	
3. Transmission control through case treatment and management	Eliminate as a public health problem	Chagas disease, cysticercosis	New anti-Chagas drugs, transmission-blocking vaccines for cysticercosis.
4. MDA and drug resistance monitoring	Regular treatment to control or reduce disease burden and morbidity	Ascariasis, trichuriasis, hookworm infection, schistosomiasis, ectoparasites	In some settings, e.g., schistosomiasis in the Caribbean, elimination possible. Vaccines for hookworm and schistosomiasis under development.
5.1. Transmission control through vector control	Reduce biological behavioral and environmental risk factors for transmission and replication	Chagas disease, dengue, leishmaniasis, plague, bartonellosis	In some settings, elimination possible. Vaccines for dengue, leishmaniasis, and leptospirosis under development.
5.2. Transmission control through control of zoonotic animal reservoir hosts	Reduce biological, behavioral and environmental risk factors for transmission and replication	Chagas disease, fascioliasis, cysticercosis, echinococcosis, leishmaniasis, leptospirosis, trichinellosis	Transmission-blocking vaccines under development for Chagas disease, cysticercosis, echinococcosis, leishmaniasis, and fascioliasis.
5.3. Transmission control through more specialized prevention and control interventions	Reduce biological, behavioral and environmental risk factors for transmission and replication	Yaws/syphillis (non-venereal), larva migrans, myiasis, superficial mycoses, Buruli ulcer, hantavirus, and viral hemorrhagic diseases	

Modified from Ault [8].

Countries in the LAC region are exploring different and novel platforms for the integrated delivery of NTD health services, and are synergizing NTD control with other disease control efforts and programs. For example, supported by grants from USAID and the Gates Foundation, Haiti is implementing a pilot project to combine MDA for LF elimination and STH control, while the Honduras Ministry of Health is piloting studies to add deworming for STH control to its maternal and child health, vitamin A delivery, and Chagas disease vector control programs. In the Dominican Republic, the Ministry of Health has successfully integrated MDA into its primary health care system in the southwest region [80]. Similarly, in Nicaragua, deworming is conducted in conjunction with annual nationwide child vaccination campaigns, and in Ecuador multiple partners collaborate to implement deworming of families as a part of a nutritional outreach program targeting the poorest communities. In Argentina, Brazil, Peru, and other countries, in technical collaboration with PAHO, Chagas disease integrated control programs now include blood bank screening, residual insecticide treatment of vector-infested homes, health promotion and education, and community surveillance and reporting of house re-infestation [49], with plans underway to add screening and treatment to prevent congenital Chagas disease transmission. To date, these innovations are relatively new, but it is expected that they will be adopted and scaled in other LAC countries in the coming years.

## Future Trends: The Inter-Sectoral Approach for Sustainable NTD Control

In recognition of the severe NTD disease burden in the Americas, the PAHO/WHO, together with the LAC governments, their national disease control programs, the US Centers for Disease Control and Prevention, and the Global Network for Neglected Tropical Diseases [1] will be embarking on efforts to further control, or in some cases, eliminate the region's NTDs. To this end, a strategic plan is being developed, which will be important to MDG targets for health and sustainable poverty reduction in LAC by 2015 [81] (Table 10). The plan uses the existing epidemiological data and adapts an ecosystems approach (Tables 8 and 9) in order to define the most effective interventions that will tackle the multifactoral determinants responsible for the persistence of NTDs, placing health within the context of social and economic development and key MDGs [72].

**Table 10:** Vision, Goal, Purpose and Scope of a Proposed Strategic Plan Framework for NTD Control.

**Vision**	Latin America and the Caribbean free of the neglected diseases that contribute to poverty and poor quality of life and health.
**Goal (development objective)**	To contribute to poverty reduction and the improvement of the health status and quality of life of the excluded and neglected populations in LAC by reducing the burden and stigma of neglected diseases by year 2015.
**Purpose (immediate objective)**	To prevent, control, or eliminate where possible neglected diseases and their stigma in excluded and neglected populations in LAC by 2015 through integrated multi-disease, inter-programmatic, and inter-sectoral approaches with local empowerment and community participation.
**Scope**	This Framework provides a roadmap to 2015 and comprehensive implementation options through 2010 for the countries of Latin America and the Caribbean to make integrated and coherent decisions pertaining to the prevention, control, and elimination of neglected diseases.

## 

Rather than a strictly disease-centered approach [4],[9],[72],[82], comprehensive public policies aimed at community development and poverty reduction will be adopted. These policies will be then implemented at the local level through the mobilization and involvement of various agencies [9],[72] under the responsibility of different government sectors (inter-sectoral action) so that they can come together in a synergistic and synchronic manner. Together with strong social participation and appropriate technologies, the inter-sectoral action completes the three pillars advocated in a primary health care strategy and will contribute towards health systems strengthening. Examples of such inter-sectoral partnering were recently reviewed [72],[82]. The collateral benefits of NTD control and elimination also provide multiple entry points for linking diverse programs and projects within the health sector and among different sectors [9],[72],[82], and could build on several PAHO/WHO and World Bank initiatives to combat disease and deprivation [8],[9]. A new generation of control tools, including vaccines, some under development by product development partnerships that include research, development, and manufacturing institutions in LAC, would represent additional innovations to fold into these infrastructures [83]. Among them are new drugs and human vaccines for dengue, hookworm (and hookworm–schistosomiasis co-infections), leishmaniasis, and leprosy [76],[84],[85], as well as transmission-blocking interventions for Chagas disease, cysticercosis, fascioliasis, leptospirosis, and leishmaniasis, which target major animal reservoir hosts [48],[84]. Overall, the control and/or elimination of NTDs represent a highly cost-effective mechanism for providing new investment opportunities in areas currently plagued with these diseases and freeing up their economic potential, i.e., for tourism and ecotourism, ecologically sound mining and oil exploration, infrastructure for rural community transportation, and sustainable crop production (traditional and non-traditional crops). The treatment and prevention of the NTDs have also been revealed as an ethical imperative to respond to the fundamental human right to health [86], particularly for LAC's poorest people, its indigenous populations and people of African descent.
